# Canadian medical tourism companies that have exited the marketplace: Content analysis of websites used to market transnational medical travel

**DOI:** 10.1186/1744-8603-7-40

**Published:** 2011-10-14

**Authors:** Leigh Turner

**Affiliations:** 1 Center for Bioethics, University of Minnesota, Minneapolis, USA (55419), USA

**Keywords:** medical tourism, Canada, cross-border healthcare, globalization, websites

## Abstract

**Background:**

Medical tourism companies play an important role in promoting transnational medical travel for elective, out-of-pocket medical procedures. Though researchers are paying increasing attention to the global phenomenon of medical tourism, to date websites of medical tourism companies have received limited scrutiny. This article analyzes websites of Canadian medical tourism companies that advertised international healthcare but ultimately exited the marketplace. Using content analysis of company websites as an investigative tool, the article provides a detailed account of medical tourism companies that were based in Canada but no longer send clients to international health care facilities.

**Methods:**

Internet searches, Google Alerts, searches on Google News Canada and ProQuest Newsstand, and searches of an Industry Canada database were used to locate medical tourism companies located in Canada. Once medical tourism companies were identified, the social science research method of content analysis was used to extract relevant information from company websites. Company websites were analyzed to determine: 1) where these businesses were based; 2) the destination countries and medical facilities that they promoted; 3) the health services they advertised; 4) core marketing messages; and 5) whether businesses marketed air travel, hotel accommodations, and holiday excursions in addition to medical procedures.

**Results:**

In total, 25 medical tourism companies that were based in Canada are now defunct. Given that an estimated 18 medical tourism companies and 7 regional, cross-border medical travel facilitators now operate in Canada, it appears that approximately half of all identifiable medical tourism companies in Canada are no longer in business. 13 of the previously operational companies were based in Ontario, 7 were located in British Columbia, 4 were situated in Quebec, and 1 was based in Alberta. 14 companies marketed medical procedures within a single country, 9 businesses marketed health care at 2 or more destination nations, and 2 companies did not specify particular health care destinations. 22 companies operated as "generalist" businesses marketing many different types of medical procedures. 3 medical tourism companies marketed "specialist" services restricted to dental procedures or organ transplants. In general, medical tourism companies marketed health services on the basis of access to affordable, timely, and high-quality care. 16 businesses offered to make travel arrangements, 20 companies offered to book hotel reservations, and 17 medical tourism companies advertised holiday excursions.

**Conclusions:**

This article provides a detailed empirical analysis of websites of medical tourism companies that were based in Canada but exited the marketplace and are now inoperative. The article identifies where these companies were located in Canada, what countries and health care facilities they selected as destination sites, the health services they advertised, how they marketed themselves in a competitive environment, and what travel-related services they promoted in addition to marketing health care. The paper reveals a fluid marketplace, with many medical tourism companies exiting this industry. In addition, by disclosing identities of companies, providing their websites, archiving these websites or print copies of websites for future studies, and analyzing content of medical tourism company websites, the article can serve as a useful resource for future studies. Citizens, health policy-makers, clinicians, and researchers can all benefit from increased insight into Canada's medical tourism industry.

## Introduction

The phrase "medical tourism" is often used to describe individuals travelling for health care and paying out-of-pocket for elective medical procedures [1-8]. Medical tourism can involve travel within the borders of the country inhabited by a particular patient [9]. However, the term usually refers to transnational travel organized for the purpose of receiving medical care [10]. "Medical tourism" sometimes refers to regional health-related travel across borders dividing one country from another. For example, Canadians seeking health care and travelling from Ontario or Québec to Michigan or Vermont are occasionally described as medical tourists [11]. More commonly, the phrase is used to describe long-distance travel such as when a resident of Canada travels to India for medical care [12]. Individuals requiring emergency treatment seek immediate access to local hospitals and clinics. In contrast, medical tourism generally involves travel for elective, non-urgent medical interventions such as hip and knee replacements, dental procedures, and spinal surgery [13]. Patients might desire prompt access to these interventions but the treatments do not fall into the category of emergency care for life-threatening health conditions. Travel to spas, resorts, hot springs, and healing retreats is often characterized as "health tourism", "wellness tourism", and "spa tourism" [14,15]. The phrase "medical tourism" is usually reserved for trips involving diagnostic tests and medical procedures falling within the scope of biomedicine. This distinction is not absolute. Many individuals combine different healing modalities and when travelling to international medical facilities seek both biomedical procedures and local healing traditions.

Travel for the purpose of obtaining medical care occurs for many different reasons and must be examined within the context of individual patient circumstances and large social-structural forces or political economies shaping access to health care [16]. For example, some uninsured residents of the U.S. travel to India and Mexico for medical care. Such individuals are often labeled medical tourists but other commentators, noting these travellers' lack of health insurance and inability to gain access to affordable care at local medical institutions, describe them as "medical refugees" or "medical exiles" [17].

Distinguishing among different types of health-related travel, researchers identify various kinds of medical tourists. Individuals travelling for kidney and liver transplants, for example, are sometimes given the label "transplant tourists" [18]. Patients seeking stem cell injections at clinics based in China, India, Mexico, Russia, and elsewhere are often called "stem cell tourists" [19]. The term "reproductive tourist" is used to describe individuals travelling for in vitro fertilization, other types of reproductive technologies, and commercial surrogacy [20]. Some scholars even use the term "suicide tourist" to describe individuals who travel to Switzerland for assistance in suicide [21]. Several of these labels are rather jarring and researchers disagree about the adequacy of various terms used to describe different types of cross-border medical travel. The primary objection to the medical tourism label is the linkage of medical procedures with the holiday-going, pleasure-seeking, leisure activities, and relaxation that the word "tourism" signifies to many individuals [22]. Though disagreement persists about what terms are best regarded as marketing labels and which phrases ought to be used by researchers, the phrase "medical tourism" is now routinely used in newspaper articles, policy documents, guidelines for patients, and peer-reviewed publications.

The subject of medical tourism attracts the attention of researchers from numerous disciplines. Scholars from anthropology, bioethics, cultural studies, law, public health, sociology, women's studies, and other fields have all made important contributions to the subject. Health law scholars provide insight into legal issues associated with cross-border medical travel [23]. Economists examine benefits and harms associated with increased international trade in health services [24,25]. Bioethicists identify ethical issues and policy concerns they associate with medical tourism and the emergence of an inadequately regulated global marketplace in health services [26,27]. In particular, they raise questions about quality of care and patient safety, continuity-of-care, commercialization of health care, public health considerations, health equity, and commodification of bodies of individuals selling organs or engaging in commercial surrogacy. Though previous research examining the phenomenon of medical tourism displays interest in how medical travel is situated in particular social, political, and economic contexts, many scholars note the need for more richly detailed, empirically-informed studies that address such topics as why patients travel for care, what types of procedures they undergo, where they travel, what quality of care they receive when they travel to international medical destinations, and what happens if they experience complications after receiving care abroad and then return to their local communities [28,29].

## Background

The study of medical tourism companies, because of their intermediary role in facilitating medical travel, is an important component of the turn toward more empirically-informed and socially situated studies of medical tourism. To date, researchers studying medical tourism companies have paid particular attention to medical tourism companies based in the United States [30-32]. Journalists mention the existence of various medical tourism companies in Canada, and several articles in peer-reviewed publications identify different medical tourism companies in Canada, but there is limited scholarly research examining the medical tourism industry in Canada [33-35]. This article responds to a gap in scholarship and seeks to promote increased understanding of medical tourism in Canada. The text provides a detailed account of where defunct Canadian medical tourism companies were based, the destination sites they marketed to clients, the health services they promoted, and what they advertised in addition to medical procedures. This topic was addressed by compiling, between 2006 and 2011, a database of medical tourism companies based in Canada. The database contains companies marketing health services at international health care facilities and functioning as medical tourism agencies. It does not include websites that simply provide information about medical tourism. Once a database of medical tourism companies was established, content analysis of company websites was used to: 1) identify and document where in Canada particular medical tourism companies are based; 2) identify and summarize the destination countries to which these businesses indicate they send clients; 3) identify and record medical procedures, tests, and treatments marketed by these businesses; 4) identify and summarize the core marketing message of each of these companies; and 5) address whether medical tourism companies are involved in booking flights, arranging accommodations at hotels and resorts, and offering holidays at resorts, side trips, holiday excursions, visits to local landmarks and other activities often associated with the word, "tourism". The social science method of content analysis was used to address these five topics by analyzing websites of medical tourism companies. After content analysis was completed, medical tourism companies that currently market health care abroad were distinguished from defunct businesses. Medical tourism companies remaining in business are examined elsewhere. Companies that have ceased operations are the focus of this article. Unlike previous contributions to the study of transnational medical travel, the purpose of this article is to identify and analyze medical tourism companies that have exited the marketplace for transnational medical travel. In addition to its analytic function, the paper has historical value by capturing a turbulent period in the medical tourism marketplace and examining those Canadian businesses that are no longer involved in competing for clients. Medical tourism is often described as an industry with a rapidly expanding client base. This paper offers a more complicated account of the market for medical tourism by revealing that of all the medical tourism companies in Canada that could be identified over the last five years, approximately half of them are no longer in business.

## Methods

### Development of a database of medical tourism companies located in Canada

Before analyzing websites of individual medical tourism companies it was first necessary to develop a database of all medical tourism companies with head offices or affiliate offices in Canada. Several methods were used to build this database. From 2006 to 2011, repeated Internet searches using Google Search Engine played a key role in locating medical tourism companies situated in Canada. Many businesses came into existence over the course of this study and were not identifiable during initial searches for medical tourism companies in Canada. Such phrases as "medical tourism company Canada", "medical tourism agency Canada", and "medical tourist Canada" were used as search terms. Searches for newspaper articles addressing such topics as "medical tourism", "medical tourism Canada", and "cross-border healthcare Canada" provided additional leads [36-40]. Newspaper articles were located using repeated searches on Google News Canada and ProQuest Newsstand. Newspaper reports of medical tourism companies situated in British Columbia, Alberta, Manitoba, Ontario, and Quebec played an important role in tracking the emergence of medical tourism companies across Canada. Google alerts for such terms as "medical tourism Canada", "stem cell tourism Canada", "transplant tourism Canada", and "global health care Canada" also generated extensive news reportage of Canadian medical tourism companies and Canadian patients travelling abroad for care. In addition, searches of an Industry Canada website that lists federally incorporated businesses in Canada yielded three company names. However, despite repeated efforts to locate these businesses it was not possible to find company websites, news media coverage concerning these businesses, or other signs indicating that these companies successfully entered the marketplace for promoting transnational medical travel. These companies are therefore not included in the final tally of twenty-five defunct companies with identifiable websites. Once company websites were identified they were bookmarked and added to the database. To facilitate content analysis, company websites were printed in their entirety. Content analysis was performed by analyzing printed versions of company websites and by periodically revisiting the Internet websites of medical tourism companies. Electronically archived websites or printouts of all identified medical tourism companies are available for review. Readers interested in assessing the content analysis provided in this article are welcome to access the primary data of the company websites.

### Comparing the database to other lists of medical tourism companies in Canada

Internet searches resulted in the identification of several sources providing partial lists of medical tourism companies found in Canada. For example, in 2006, *Maclean's*, a popular news magazine in Canada, published an article that identified eight medical tourism companies in Canada [41]. Another list identifying five medical tourism businesses was found on a website promoting private health care alternatives and out-of-country health care options for Canadians [42]. A third source listed eight Canadian medical tourism companies within a longer record of medical tourism companies based in countries around the world [43]. Locating these lists permitted comparison of my database with other summaries of medical tourism companies based in Canada.

### Content analysis of websites of medical tourism companies

After developing the database content analysis was used to study and analyze websites of all identified medical tourism companies based in Canada. Company websites were analyzed using pre-determined analytic categories; content was extracted for each category. The social science method of content analysis, as the phrase suggests, involves studying the content of such media as magazines, newspapers, films, television programs, and interview transcripts [44]. Content can be analyzed for general themes and concepts, images, particular statements, and other features. For the purpose of this study, medical tourism company websites were searched for specific pieces of information or website content. There were five main components to content analysis.

#### Location of medical tourism companies

First, I identified the city or town and province that medical tourism company websites identified as their business address within Canada. Identifying the location of medical tourism companies typically involved finding and then selecting such website tabs as "Contact us", "Contact information", or "Address" and recording information indicating where companies are located. Gathering this information resulted in a detailed record of where in Canada now-defunct medical tourism companies were based. In particular, it helped establish where they clustered. Though medical tourism companies in some respects are based "on" the internet, the physical location of companies matters for numerous reasons. For example, companies are governed by the legislation and regulations of the specific regions in which they are located. Within Canada, companies must operate according to both federal and provincial legislation; the province in which companies are situated informs which legal standards can be applied to them.

#### Destination countries and health care facilities

Second, I recorded the destination countries and/or destination health care facilities that now-inoperative medical tourism companies once marketed to their clients. Some medical tourism company websites identified particular hospitals and clinics. Other websites mentioned destination nations or networks of countries but did not list specific medical facilities. Websites were analyzed to develop a better understanding of destinations promoted by Canadian medical tourism companies.

#### Types of advertised medical procedures and medical specialties

Third, to provide insight into the types of health care packages medical tourism companies promoted, I recorded medical procedures or specialties marketed by medical tourism companies. Some companies provided lengthy lists of procedures. Other companies identified particular areas of medicine without listing specific procedures. Other businesses mentioned clinical specializations such as cardiology and gastroenterology or listed illnesses rather than focusing upon distinct medical procedures. This topic was explored to better understand what types of tests and treatments medical tourism companies marketed to prospective clients.

#### Core marketing messages of medical tourism companies

Fourth, websites of medical tourism companies were reviewed for statements that best encapsulated each company's core marketing message. These statements were typically located on the home page of company websites or in such sections as "About us", "What We Do", or "Mission statement". Core marketing messages were recorded and then summarized in brief point form. This topic was studied to develop a detailed account of the market niche now-defunct Canadian medical tourism companies attempted to occupy while they remained operational.

#### "Tourism" component of medical tourism

Fifth, company websites were reviewed for information about involvement of these businesses in booking air and/or ground transport, reserving hotel accommodations, and organizing tours, side trips, and/or other holiday excursions. This topic was studied in an attempt to determine whether holidays, side trips and other tourism-related activities were marketed in addition to health care packages.

In most instances, websites provided detailed information for each study question or category of content analysis. Data was recorded in tabular form. Where websites did not provide pertinent information, "NA" ("Not Addressed" in abbreviated form) was noted in tables.

The author of this article performed content analysis. The paper was fact-check three times by the author and feedback was solicited from two senior colleagues. All websites are disclosed and electronically archived. Readers interested in assessing the quality of content analysis have access to the primary data.

### Distinguishing functioning from inoperative medical tourism companies

Following content analysis of all identified medical tourism company websites, in February and March 2011, internet searches, searches of an Industry Canada database [45] identifying the status of federally incorporated businesses in Canada, phone calls, and emails were used to distinguish active medical tourism businesses from medical tourism companies with expired websites, non-functioning email accounts, and discontinued phone numbers. Email and phone queries were used to determine whether businesses remained in operation. According to research ethics guidelines in both Canada, where this research project was initiated, and the United States, where it was completed, contacting a company to see if it remains in business does not fall within the scope of research requiring research ethics board review because there was no attempt to ask company owners or employees about their role within these companies, explore their attitudes toward medical tourism, investigate their workplace activities, or otherwise treat them as research subjects [46].

## Results

Using Internet searches, searches for newspaper articles mentioning medical tourism companies, and Google Alerts, it was possible to locate a total of twenty-five Canada-based medical tourism companies that have ceased sending their clients to hospitals and clinics located outside Canada. In contrast, operational medical tourism companies include an estimated 18 businesses marketing medical travel to such countries as Costa Rica, India, and Thailand and 7 businesses marketing regional, cross-border health services available in the United States as well as travel to private clinics within Canada. Several additional businesses are not comprehensive medical tourism companies but market bariatric surgery procedures performed in facilities based outside Canada as well as so-called "Liberation therapy" performed in India. Excluding these latter "boutique" businesses, approximately half of all medical tourism companies in Canada remain operational and the other half has disappeared from the marketplace.

Companies were labeled inoperative if websites were deactivated and phone lines were disconnected, company representatives reported that they were no longer accepting clients and had halted operations, they had lost their status as federally incorporated companies, or seven phone calls and/or emails failed to generate any response. It is possible that businesses that at present are not going concerns could at some point build a client base and begin sending customers to international medical facilities. If this occurs, in future analyses these companies will be reclassified as functioning medical tourism companies. My research provides a "snapshot" of Canada's medical tourism "landscape" at a particular moment; it is important to appreciate that the medical tourism industry in Canada is in flux. It is reasonable to assume that this industry is going to change over time as some companies emerge, other businesses disappear, and other companies transform themselves in response to client demand and perceived marketing opportunities.

Of the inoperative companies that I was able to locate, twenty-five had active, functioning websites at some point between 2006 and 2011. Additional file 1 identifies these businesses, provides website links, and contains Webcite references. Three additional companies, St. Luke Medical Tourism Center of North America Inc., Medical Tourism China, and International Medical Travel Corporation, were identified in the Government of Canada's database of businesses federally incorporated in Canada but do not appear to have had websites, issued press releases, or attracted any news media coverage. It is unclear whether these companies ever had clients even though they were incorporated businesses presumably established for the purpose of marketing medical tourism. These three businesses are noted but they are not included in the total tally of twenty-five defunct medical tourism companies. With no websites to analyze, and no indications that they had ever sent clients to international destinations, there was no basis for including them in the content analysis process.

### Locations of medical tourism companies

Of the twenty-five companies that established websites and were studied using the method of content analysis, thirteen were located in Ontario, seven were based in British Columbia, four were situated in Quebec, and one was located in Alberta. Websites of some Canadian medical tourism companies identify affiliate offices or company representatives located outside Canada. Where such information was provided I recorded where company representatives based outside Canada were situated.

Table 1 lists company names, identifies where in Canada these businesses were located, and documents affiliates and representatives in those instances where companies had offices or agents situated outside Canada.

**Table 1:** Locations of Medical Tourism Companies in Canada

Company	City & Province
Axiom Health Solutions	Canadian Office: Oakville, Ontario; U.S. Office: Kensigton [sic], Maryland

Canadian Healthcare International (CHI)	Markham, Ontario

CubaMedicare	Oakville, Ontario

EcuMedical Resources International Ltd.	Windsor, Ontario

First Choice Medical Tourism	Canadian Office: Calgary, Alberta; with contact person in Luzon, Philippines

Health Trips	Ottawa, Ontario

Health Vacations, Inc.	Ottawa, Ontario

International Medical Network	Oakville, Ontario

JD Healthcare	Nanaimo, British Columbia

LAM International (L.A.M. Logistic.Assistance.Medical International)	Canadian Office: Toronto, Ontario; and Colombia

MedAsia	Montreal, Quebec

MedExpress Tourism	Montreal, Quebec

Medi-Pro Medical Management	Windsor, Ontario

MedSolution	Vancouver, British Columbia

Medtourlink	Vancouver, British Columbia

Reach Health Services & Outsourcing	Canadian Office: Vancouver, British Columbia; Representative in Chennai, India

Recover Discover Healthcare	Canadian Office: Vancouver British, Columbia; U.S. Office: Austin, Texas; India Office: Noida, India

Royal Med Services	Mississauga (Toronto), Ontario

Speedy Surgery Global Healthcare	Quebec City, Quebec

Star Hospitals	Canadian marketing office in Toronto, call centers and operations in Chennai, India

Sun Medical Group	Vancouver, British Columbia

The InciDental Tourist	Ottawa, Ontario

Tooth Tourism	Surrey, British Columbia

Unbelievable India	Dollard-Des-Ormeaux, Quebec

Victus Global Healthcare	Main Office: Toronto, Ontario; Additional office: Edmonton, Alberta

### Destination countries and health care facilities

Of the twenty-five companies, fourteen marketed medical procedures at just one medical facility or at several medical facilities within one country. Three companies marketed health care in two destination nations, two companies advertised procedures in three countries, two companies promoted health services in four countries, one company marketed procedures in five countries, and one of the dental tourism companies marketed dental procedures in seven countries. Two companies did not provide details concerning where they sent their clients.

The twenty-five medical tourism companies marketed health services in twenty-one different nations. Twelve companies marketed medical travel to India; four listed Thailand as a destination site; three companies listed Singapore; Canada, Cuba, the United States, the Philippines, Costa Rica, El Salvador, China, Malaysia, and Mexico were all twice listed as destination sites; and Morocco, Russia, Hungary, Colombia, Taiwan, France, Turkey, Dominican Republic, and Panama were all listed once. Listing of countries as destinations does not mean that any residents of Canada ever selected these particular destinations as sites for medical care. However, documentation of advertised health care destinations provides insight into the partnerships or networks medical tourism companies choose to market to their prospective client base. While they do not provide information about actual flows of patients across national borders, they reveal how medical tourism companies that were based in Canada promoted health facilities in other nations. Table 2 identifies locations of destination facilities marketed on company websites.

**Table 2:** Medical Tourism Companies and Destination Facilities

Company	Destination Facilities
Axiom Health Solutions	Apollo Hospitals, Wockhardt Hospitals, Escorts Heart Institute and Research Centre, Max Healthcare, Fortis Healthcare, Sahaj Dental Clinic, all in India

Canadian Healthcare International (CHI)	Health care facilities in Canada (particular institutions are not identified)

CubaMedicare	Medical facilities in Havana, Varadero, and Holguin, all in Cuba

EcuMedical Resources International	Health care facilities in the United States

First Choice Medical Tourism	St. Luke's Medical Center, Quezon City, Philippines; Estetico Manila, Manila, Philippines

Health Trips	German Collaborative Private International Hospital, India

Health Vacations, Inc.	St. Petersburg, Russia

International Medical Network	Hospitals and clinics in India, Hungary, Cuba, and Costa Rica

JD Healthcare	Website claims company has partnerships with more than 30 hospitals (particular institutions are not specified)

LAM International (Logistic.Assistance. Medical International)	Transplant facilities in Colombia

MedAsia	Min-Sheng General Hospital, Taiwan

MedExpress Tourism	Health care facilities in Morocco

Medi-Pro Medical Management	Laser Spine Institute, Tampa, Florida; Crittenton Hospital, Rochester, MI, Internal Medicine, Mount Clemens, MI; additional facilities in the U.S.

MedSolution	India, France, Turkey, El Salvador; website indicates plan to include Costa Rica, Malaysia, Mexico, Poland, and South Africa

Medtourlink	Singapore, Philippines, India, China, Canada

Reach Health Services & Outsourcing	Apollo Hospitals, Escorts Heart Institute and Research Centre, Rajan Eye Care, JGHR Dental, all in India

Recover Discover Healthcare	Escorts Heart Institute & Research Centre, Delhi; Fortis Hospital, Noida; Fortis Flt. Lt Rajan Dhall Hospital, Delhi; Fortis Hospital, Mohali, Chandigarh; Fortis-Wockhardt Hospital, Bangalore; Fortis-Wockhardt Hospital, Mumbai, India; Gleneagles Medical Center, Penang, Malaysia

Royal Med Services	Medical facilities in India

Speedy Surgery Global Healthcare	Medical facilities in India and Thailand

Star Hospitals	India, Singapore, Thailand

Sun Medical Group	Malaysia, Singapore, Thailand

The InciDental Tourist	Nanjing,China; Merida, Mexico

Tooth Tourism	Costa Rica, Dominican Republic, El Salvador, India, Mexico, Panama, Bangkok, Thailand

Unbelievable India	Medical facilities in India

Victus Global Healthcare	JCI-accredited medical facilities around the world (particular institutions are not specified)

### Types of advertised medical procedures

Twenty-two companies marketed many different kinds of medical procedures and can be characterized as "generalist" medical tourism companies. They provide lengthy lists of different types of medical procedures. Three businesses marketed specialized services and restricted themselves to a niche position within the medical tourism industry. Of these latter businesses, two companies limited themselves to marketing dental procedures and one company advertised organ transplants in Colombia. Table 3 summarizes health services marketed by medical tourism companies.

**Table 3:** Marketed Procedures

Company	Health Services Marketed
Axiom Health Solutions	Interventional cardiology, cardiothoracic surgery, cosmetic and plastic surgery, hand and micro surgery, medical & surgical gastroenterology, neurology & neurosurgery, spine surgery, surgical oncology, orthopedic surgery, vascular surgery, brain surgery, bone and joint surgery, eye surgery, minimal access surgery, obesity surgery, maxillofacial surgery, thoracic surgery, orthopedic surgery, dental care and dental surgery, transplant surgery, and other treatments

Canadian Healthcare International (CHI)	Specific procedures are not identified though total hip replacement and arthroscopic rotator cuff repair are used to compare cost of care in Canadian facilities versus institutions in other countries

CubaMedicare	Drug and alcohol rehabilitation, pigmentary retinosis, neurological rehabilitation, vitiligo, psoriasis, and alopecia, cosmetic surgery, odontology, dental implants, orthognatic surgery; alternative therapies, stroke rehabilitation, spinal cord injury rehabilitation, brain injury rehabilitation, physical, occupational, and speech therapy, aquatic therapy, physical therapy, anorexia nervosa treatments

EcuMedical Resources International Ltd.	cancer, cardiology, cosmetic surgery, day surgery, diagnostic imaging, hip surgery, knee surgery, minimally invasive surgery, neck and spine surgery, neurology, orthopaedics, second opinions, urology, VIP services

First Choice Medical Tourism	General medical surgery, dental surgery, cosmetic surgery, optical surgery, spa retreats

Health Trips	knee replacement, hip replacement, shoulder replacement, spinal surgery, correction of deformities, beating heart surgery, cosmetic surgery

Health Vacations, Inc.	MRI outsourcing, endocrinology surgery, orthopedic packages, ophthalmology packages, spinal surgery, diagnosis and treatment of allergies, preventive health check ups, executive health check ups, preventive heart checkup, whole body check up, and additional treatments, access to specialists in cardiology, internal medicine, neurology, obstetrics/gynecology, orthopedic surgery, pediatrics, urology, endocrinology, rheumatology

International Medical Network	Cardiovascular, orthopedic, neurosurgery, female reproductive system, gastro-intestinal, cosmetic, dental, IVF

JD Healthcare	Assisted reproduction, plastic surgery, bone and joint surgeries, cancer treatment, cardiovascular surgery, eye surgery, general surgery, cerebrovascular surgery, dental surgery, dermatology, and additional treatments

LAM International (Logistic. Assistance. Medical International)	Heart transplants, liver transplants, lung transplants, kidney transplants

MedAsia	Bariatric surgery, cardiovascular surgery including minimally invasive cardiovascular surgery, PTCA and stent, and cardiac catheterization, orthopedic surgery including total joint replacement, spine surgery, and arthroscopy, other treatments

MedExpress Tourism	Dental implants, weight loss, cosmetic surgery, orthopedics, urology, cardiology, eye corrective (Lasik), cancer, general surgery

Medi-Pro Medical Management	MRI scans, CT scans, body imaging, X-rays, arthroscopy, bariatric surgery, cardiology, endocrinology, internal medicine, neurology

MedSolution	Cardiac care, chemical dependency programs, cosmetic & plastic surgery, dental care, gastric surgery, heart surgery, infertility treatments, orthopedic surgery, urogenital surgery

Medtourlink	Hysterectomy, cataract, Lasik eye care procedures, breast lift, tummy tuck, facelift, rhinoplasty, dental implants, dentures, crowns and aesthetic dentistry, MRI, X-ray, PET, CT diagnostic scans, laser procedures

Reach Health Services & Outsourcing	Cardiac surgery and cardiology, minimal access surgery and urology, orthopedics, ophthalmology, gastroenterology, obstetric and gynecology, oncology, cosmetic/plastic surgery, preventive health checks, diagnostic services

Recover Discover Healthcare	Knee replacement surgery, hip replacement surgery, hip resurfacing, adult & pediatric cardiac care, oncology treatments, bariatric care, laparoscopic gastric band, gastric bypass surgery, sleeve gastrectomy, neurosurgeries, spinal care, infertility management including IVF, cosmetic care, additional surgeries

Royal Med Services	Hip replacements, other joint replacements, breast augmentation, liposuction, and other procedures

Speedy Surgery Global Healthcare	Allergy, cardiology, dentistry, diabetes and endocrinology, ear, nose and throat diseases, eye laser refraction, oncology, ophthalmology, orthopedic, plastic surgery, preventive health check, radiology and imaging services, and additional procedures

Star Hospitals	cardiology, cardiothoracic surgery, orthopedic, neurology and neurosurgery, eye care, cosmetic surgery, dentistry, comprehensive and preventive health checks, weight loss, obstetrics and gynecology, IVF, additional treatments, yoga, Ayurvedic consultation, stress management, weight reduction, spine and joint care

Sun Medical Group	Dental surgery, eye care, cardiac interventions, orthopaedic surgery, cosmetic surgery

The InciDental Tourist	bridgework, partial and full crowns, laser tooth bleaching, implants, inlays, onlays, veneers, root canals, extractions, and other dental procedures

Tooth Tourism	Dental bonding and contouring, dental bridge, dental crowns, dental fillings, dental implants, dental veneers, dentures, gingivectomy, root canal, teeth whitening

Unbelievable India	Plastic surgery, correction of congenital malformations, reconstructive surgery, orthopedics, ophthalmology, hip replacements, Ayurvedic medicine, dental procedures, other treatments

Victus Global Healthcare	Heart bypass, heart valve replacement, hip replacement, hysterectomy, knee replacement, spinal fusion, additional treatments

### Core marketing messages of medical tourism companies

Though there were variations in core marketing messages of medical tourism companies, most businesses emphasized affordability of care, timely access to medical care, and high-quality care. In total, of twenty-five businesses, twenty companies marketed access to affordable care, seventeen businesses advertized timely access to care, and twenty-one websites emphasized high-quality care. The few companies that did not mention all three features offered a subset of these offerings.

Table 4 provides summaries of medical tourism companies' core marketing messages. Additional file 2 provides summaries of core marketing messages in addition to extended excerpts from company websites. These lengthy excerpts are intended to demonstrate the empirical basis for identifying and summarizing core marketing messages.

**Table 4:** Summary of Core Marketing Messages

Company	Core Marketing Message
Axiom Health Solutions	access to affordable, timely, and high-quality care

Canadian Healthcare International (CHI)	access to affordable and high quality health care in Canada

CubaMedicare	access to affordable, timely, and high quality health care as well as vacation experience

EcuMedical Resources International Ltd.	access to timely and high-quality health care

First Choice Medical Tourism	access to affordable, timely, and high quality health care as well as holiday experience

Health Trips	access to affordable and high-quality care as well as vacation experience

Health Vacations, Inc.	access to affordable, timely, and high-quality care

International Medical Network	access to affordable, timely and high-quality care

JD Healthcare	access to affordable, timely and high-quality health care

LAM International (Logistic.Assistance.Medical International)	access to timely organ transplants

MedAsia	access to affordable, timely, and high-quality care

MedExpress Tourism	access to affordable and high-quality care

Medi-Pro Medical Management	access to timely and high-quality care

MedSolution	access to affordable, timely and high-quality care

Medtourlink	access to high-quality care

Reach Health Services & Outsourcing	Access to affordable and timely health care

Recover Discover Healthcare	access to affordable and high- quality care

Royal Med Services	access to high-quality health care

Speedy Surgery Global Healthcare	access to affordable, timely and high-quality health care

Star Hospitals	access to affordable, timely, and high-quality health care

Sun Medical Group	access to affordable and timely care

The InciDental Tourist	access to affordable and high-quality care

Tooth Tourism	access to affordable and high-quality dental care

Unbelievable India	access to affordable, timely, and high-quality care

Victus Global Healthcare	access to affordable and timely health care (messages targeted at employers rather than individuals)

### "Tourism" component of medical tourism

Core marketing messages rarely emphasized the possibility of patients undergoing medical procedures while also enjoying holiday excursions. However, activities commonly associated with the concept of tourism were marketed by more than half of the companies. Sixteen medical tourism companies offered to arrange air travel and/or organize ground transport. Twenty companies marketed the service of booking hotel reservations. Seventeen businesses advertised tours, side trips, and other holiday excursions in addition to medical care. This latter finding will not resolve disagreements about whether "medical tourism" is a suitable term for academic analysis. However, it does reveal that many medical tourism companies in Canada market both medical care and leisure or "holiday" activities commonly associated with tourism. Table 5 identifies whether medical tourism companies booked travel arrangements, arranged hotel accommodations, and offered holiday excursions, side trips, visits to local attractions, and other "tourist-like" excursions.

**Table 5:** Medical Tourism Companies Marketing Travel, Accommodations, and Tourism-related Activities (Y = Yes, N = No, NA = Not Addressed on company website)

Company	Book Travel	Book Hotel	Book Tours
Axiom Health Solutions	Y	Y	Y

Canadian Healthcare International (CHI)	Y	Y	NA

CubaMedicare	Y	Y	Y

EcuMedical Resources International Ltd.	Y	Y	NA

First Choice Medical Tourism	NA	NA	Y

Health Trips	Y	Y	Y

Health Vacations, Inc.	Y	Y	Y

International Medical Network	Y	Y	Y

JD Healthcare	N	NA	Y

LAM International (Logistic.Assistance.Medical International)	NA	Y	Y

MedAsia	Y	Y	Y

MedExpress Tourism	Y	Y	N

Medi-Pro Medical Management	Y	NA	NA

MedSolution	Y	Y	Y

Medtourlink	NA	Y	NA

Reach Health Services & Outsourcing	NA	Y	NA

Recover Discover Healthcare	NA	NA	Y

Royal Med Services	Y	Y	Y

Speedy Surgery Global Healthcare	Y	Y	NA

Star Hospitals	Y	Y	Y

Sun Medical Group	NA	NA	Y

The InciDental Tourist	N	Y	Y

Tooth Tourism	N	Y	Y

Unbelievable India (partnership with Focuz Group)	Y	Y	Y

Victus Global Healthcare	Y	Y	NA

## Discussion

### Principal Results

#### Value in studying functioning and inoperative medical tourism companies

To date, there are no published studies that identify by company name, analyze in systematic fashion, disclose in transparent manner, and permanently archive websites of medical tourism companies with head offices or affiliate offices in Canada. Previous articles mention several medical travel facilitators based in Canada but do not provide a comprehensive overview of identifiable medical tourism companies, disclose their websites, analyze their business operations, and clearly distinguish between functioning and defunct companies [47]. This article provides insight into those medical tourism companies in Canada that came into existence and at some point ceased operations between 2006-2011. To some individuals, identifying and analyzing medical tourism companies that have exited the marketplace might seem like an unproductive exercise. However, identifying and analyzing medical tourism companies that have ceased functioning serves two purposes. First, it contributes to the overall analysis of Canada's medical tourism industry rather than presenting just a limited account of businesses successfully involved in advertising health care at international destinations. Analyzing companies that ceased functioning permits insight into where they were based, what health care procedures they marketed, where they proposed sending clients, what part of the marketplace they sought to occupy, and whether they advertised tourism-related activities such as offering side trips and holiday excursions in addition to booking flights and accommodations. Second, and perhaps more importantly, identifying and examining inoperative medical tourism companies challenges the hyperbolic marketing rhetoric surrounding the topic of medical tourism and could play a role in promoting more balanced ethical, social, and economic analysis of the subject [48]. Not all medical tourism companies remain going concerns. Studying websites of medical tourism companies provides no insight into how many Canadians seek care beyond Canada's borders. Nonetheless, perhaps the disappearance of an estimated 50% of all medical tourism companies based in Canada should prompt questions about the overall size of the marketplace within Canada for international medical travel. It is conceivable that the market for medical care abroad is smaller than anticipated and some medical tourism companies fail because their prospective clientele is less numerous than assumed [49]. Another possibility is that of the many medical tourism companies established in Canada, only a limited number of them have successfully developed significant client bases. It is also conceivable that some medical tourism companies were commercially successful but closed for non-financial reasons. This study does not attempt to address these questions but it should prompt critical reflection on the size and evolving shape of Canada's marketplace for medical tourism. Future research will attempt to address the question of why some medical tourism companies exit the marketplace whereas other businesses appear to be successful at attracting clients and expanding their operations.

Key findings from analysis of websites of Canadian medical tourism companies are that twenty-five companies established for the purpose of sending residents of Canada to medical facilities in other nations are no longer operational, most defunct medical tourism companies are "generalist" rather than "specialist" businesses, and many companies offer tourism-related activities in addition to marketing medical procedures.

#### Medical tourism company "hotspots"

Of the twenty-five companies with websites, thirteen businesses based in Ontario are now defunct, seven companies in British Columbia ceased operations, four companies in Quebec are closed, and one company in Alberta did not respond to calls and emails and is presumed closed. This study does not address how many individuals travel from Canadian provinces to international medical destinations. However, it is worth noting the concentration of defunct medical travel companies in Ontario, British Columbia, and Québec. These provinces also happen to be the three provinces with the greatest number of medical tourism companies still functioning. Perhaps they should be characterized as "hotspots" for both the establishment and closure of Canadian medical tourism companies. Future research will explore why numerous medical tourism companies have been established in some Canadian provinces whereas other provinces have no businesses involved in the medical tourism industry.

#### Choice of marketing messages

There likely are numerous reasons why medical tourism companies based in Canada use particular marketing messages. Most clients of these businesses must pay out-of-pocket for private medical care provided by hospitals and clinics based outside Canada. Though there are some circumstances in which provincial health insurance plans will fund provision of elective medical procedures performed outside Canada, in most instances clients of these businesses must use personal savings to purchase treatment. It is therefore unsurprisingly that affordability of care is emphasized in marketing messages. Access to high-quality care is presumably mentioned as a way to reassure prospective clients that they should feel confident that they will receive professional care when they go abroad for treatment. This message is perhaps promoted as a way of allaying fears that going abroad for inexpensive care might increase risk of exposure to lower-quality care. Finally, the message of gaining access to "timely" health care might have particular resonance for Canadians wait-listed for orthopaedic procedures, ophthalmologic procedures, spinal surgery, bariatric surgery, and other interventions where there are sometimes considerable delays prior to obtaining access to publicly-funded health care at Canadian medical facilities. For example, Speedy Surgery Global Healthcare, one defunct company mentioned in this analysis, and Timely Medical Alternatives, a company that continues sending Canadians to hospitals and clinics in the United States as well as private health care facilities in Canada, both emphasize in their company names provision of prompt access to care. Wanting to avoid providing a reductionist, single-factor account of how medical tourism is marketed by companies based in Canada, it nonetheless seems reasonable to suggest that timely access to care abroad, in contrast to treatment delays for access to some types of care in medical procedures performed in Canada, is one marketing message likely to resonate with Canadians frustrated with wait-times for various elective procedures. In this respect, it is possible that the marketing messages used by medical tourism companies based in Canada might differ somewhat from marketing claims used by medical tourism companies located in countries where the presence of a significant private health sector means that clients with sufficient financial assets can gain prompt access to medical procedures. Comparative studies that contrast marketing messages of medical tourism companies based in different countries should provide insight into regional and national variations in the types of marketing messages used to promote medical tourism.

#### Generalist and specialist companies

Another finding that merits mention is that twenty-two of the twenty-five defunct medical tourism companies can be characterized as "generalist" medical tourism companies. This label means that these companies marketed multiple procedures, tests, and treatments rather than confining themselves to dental treatments or some other specialized domain of care. Even companies that offer many different services are at risk of ceasing operations. It is not known whether cessation of operations is linked to a limited client base, an overreliance on one or two health care destinations, concerns about liability, competition from other medical tourism companies, a low ratio of earnings to expenditures, or other factors. Future research will explore why some companies remain operational and other businesses cease functioning.

#### Closure of dental tourism companies

Both of the companies that promoted "dental tourism" are no longer operational. While this article does not address why medical tourism companies ceased operations, numerous hypotheses are worth exploring in future studies. Cost savings associated with obtaining dental care abroad might be lower than anticipated once airline tickets, ground transport, hotel accommodations, and other expenses are factored into consideration. Concerns about patient safety and quality of care might deter some Canadians from receiving dental care abroad. It is also conceivable that the practice of dentistry is not well suited to a business model that involves travel to international dental clinics. Many forms of dental care require repeat visits rather than one-time procedures. Having multiple dental procedures at one time could put patients at increased risk of complications. Staying abroad for multiple procedures would presumably increase time away from home and also generate additional expenses. Though this study offers no definitive insights into why both of Canada's dental tourism companies are defunct, it is worth noting that at present there appear to be no functioning companies in Canada that are based exclusively upon a business model of marketing dental care abroad.

#### Little marketing of organ transplants performed abroad

Travel abroad for the purpose of purchasing a kidney used in organ transplantation, sometimes described as "transplant tourism", receives considerable attention from both health researchers and journalists. However, of Canadian medical tourism companies no longer in operation, just one business had as its primary message the marketing of organ transplants abroad. One other company marketed organ transplants in addition to other medical procedures. It is conceivable that other medical tourism companies did not market organ transplants abroad because of fears that they might be accused of facilitating "organ brokering" or "organ trafficking"; concerns about negative news media coverage that might harm other aspects of their business; inability to identify facilities able to provide organ transplants; ethical reservations about intentionally or unintentionally participating in organ trafficking; or concerns about legal repercussions. Whatever the reason, with the exception of one business that advertised organ transplants in Colombia and one company that claimed to be able to arrange transplants in India, most now-defunct medical tourism companies in Canada did not, while they remained operational, openly market organ transplants performed in other countries.

#### Medical consumerism and restrictions on choice of destination nations

Another noteworthy finding is that of the twenty-five businesses with websites, fourteen of them sent their clients to just one medical facility or destination nation. In this business model, owners of medical tourism companies decide that all their clients will travel to just one location. It is conceivable that this arrangement is not an optimal business model for an industry that in many respects is built upon promoting patient choice and medical consumerism. It is possible that medical tourism companies offer more attractive options to a larger prospective clientele when they market health care facilities at multiple international destinations. Whether or not this claim is credible, it is important to note that even businesses with multiple destinations ceased operations. One dental tourism company, for example, listed seven national destinations on its website. In short, while limiting the number of medical destinations constrains choice and possibly acts as a deterrent to prospective clients, promoting choice in the form of multiple travel destinations does not ensure long-term success in the medical tourism marketplace.

#### "Tourism" component of medical tourism

Though some researchers express reservations about the phrase "medical tourism", many now-inoperative medical tourism companies in Canada marketed travel-related services such as booking air and ground transport, organizing hotel accommodations and arranging side trips, and other tourist-like activities. In this aspect of their business models, medical tourism companies resemble travel agencies. These activities provide some insight into how medical care can be connected to activities more typically associated with "traditional" forms of tourism.

#### Limited media coverage of defunct medical tourism companies

Whatever causes medical tourism companies to close, it is important to note that in Canada a substantial number of medical tourism companies have exited the marketplace. The closure of approximately half of all medical tourism companies in Canada has not been previously examined and addressed by health researchers and journalists. Whereas medical tourism companies routinely issue press releases and attract news media coverage when they commence operations, these businesses typically do not issue press releases or receive news coverage when they close. Just one business, EcuMedical Resources International, generated news coverage as it ceased functioning [50,51]. In that company's case, several of its clients claimed that they paid EcuMedical for arranging medical procedures in the U.S. and then began receiving bills from the hospitals where they received care. Following a police investigation the two company owners were charged with multiple counts of fraud. In this case, activity that is presumably atypical in the medical tourism industry resulted in considerable local news coverage of a medical tourism company ceasing operations.

### Basis for additional research

One important benefit of studying medical tourism companies by analyzing content of medical tourism company websites is that the exercise can challenge hitherto unexamined assumptions about the industry. More fine-grained analyses that draw upon multiple research methods such as surveys, in-depth interviews, analysis of news media coverage, and participant observation should generate additional insights into medical tourism companies in Canada. For example, quantitative studies should provide insight into how many Canadians go abroad for care, where they seek treatment, and what types of procedures they select. Identifying Canada's medical tourism companies, providing their websites, describing their operations and business models, charting where they send patients abroad, summarizing the procedures they advertise, and explicating what services they offer in addition to organizing health care packages should facilitate further studies into Canada's medical tourism industry. It should also be of practical use to scholars interested in comparing medical tourism companies in Canada with similar businesses situated in other countries. For example, researchers in Australia, England, the United States and elsewhere might find it helpful to compare and contrast Canada's medical tourism industry with the emergence of medical tourism facilitators in their home countries. In addition, these findings should assist scholars interested in evaluating the quality of health-related information presented on the websites of medical tourism companies, and the use of YouTube, Twitter, Facebook, and various forms of social media by medical tourism companies situated in Canada. Scholars interested in using various research methods to study medical tourism companies in Canada should now have a better sense of where to focus their attention. To facilitate research by other scholars, websites or printouts of websites analyzed in this article have been electronically archived in a publicly accessible database.

### Limitations

#### Findings limited to medical tourism companies based in Canada

This article is based upon content analysis of websites of medical tourism companies with home offices or affiliate offices in Canada. It does not provide insight into medical tourism companies located in other nations. This study offers a set of categories or framework that can be used to study medical tourism companies in other nations. However, using content analysis to study websites of medical tourism companies based outside Canada might generate results very different from this study's findings.

#### Possibility of undiscovered websites

Internet searches, review of news reports, review of government records of federally incorporated companies in Canada, and examination of existing lists of medical tourism companies in Canada were all used in an attempt both to identify the total number of medical tourism companies established in Canada and distinguish medical tourism companies that are currently functioning from companies that are no longer involved in sending clients abroad for medical care. However, it is possible that some medical tourism companies were not identified in these searches. Medical tourism companies use company websites, Twitter, YouTube, Facebook, and other media to market international care, and internet searches are therefore a useful tool to use when trying to find medical tourism companies. Nonetheless, it is conceivable that some medical tourism companies based in Canada do not advertise using the internet or that repeated searches overlooked some businesses.

#### Possibility that non-responders might remain in business

Companies that did not respond to seven phone calls and/or emails over a three week period of repeated queries were listed as defunct. Consultants to the medical tourism industry recommend that calls to medical tourism companies should be promptly returned within twenty-four hours and preferably within two hours [52]. However, it is possible that some medical tourism companies continue to function even though they did not reply to multiple efforts to contact them. Establishing that businesses have ceased functioning in the absence of obvious signs such as companies losing their status as federally incorporated businesses in the end involved exercising judgement.

#### Investigative research methods might challenge claims made by medical tourism company websites

The study is limited insofar as it analyzes content found on medical tourism company websites and does not question the veracity of various claims. For example, one medical tourism company website states that clients can select clinics from among seven different countries. Someone using the tools of investigative journalism might discover that companies do not have business relations with all the facilities that they mention as international health care destinations. Such questions were bracketed from this study but they are worth pursuing in other studies. It is conceivable that some companies make exaggerated claims about destination facilities and advertised medical services and do not have experience coordinating particular health care packages and sending clients to advertised destinations. Content analysis, for the purpose of this article, accepted statements made by medical tourism companies. Such an approach, though it provides insight into how medical tourism companies in Canada advertise their services, would benefit from being complemented by investigative approaches that critically examine the credibility of claims made by medical tourism companies.

#### Studying Defunct Companies

This article focuses upon medical tourism companies that ceased operations. Performing a content analysis of websites of defunct companies could be seen as a limitation of the study, though the point of the exercise was to investigate and analyze not only medical tourism companies that are flourishing in a highly competitive marketplace but also to analyze websites of companies that have ceased sending clients to international health care destinations.

### Comparison With Prior Work

#### Focus on Canada

To date, most studies of medical tourism companies have focused upon websites of medical tourism facilitators based in the United States. This paper makes a meaningful contribution to scholarship by providing content analysis of medical tourism companies with home offices or affiliate branches in Canada. The local, regional, and national settings within which medical tourism companies are situated matters. It should not be assumed that medical tourism companies in Canada advertise the same health care packages or target the same prospective client base as businesses in Australia, the United States, and elsewhere.

#### Disclosure of Primary Sources

This article differs from previous work on medical tourism companies by identifying particular businesses, performing content analysis of their websites, and enabling readers to compare this analysis with the primary sources of medical tourism company websites. This step, by linking content analysis to identified primary sources, promotes transparency in research and allows other scholars to corroborate or challenge findings described in this study. Identifying particular companies and providing content analysis of their websites might help other researchers use their disciplinary methods and theories to study Canada's medical tourism industry.

#### Analyzing Company Websites

This article can be distinguished from most prior work on medical tourism due to its emphasis on content analysis of medical tourism company websites. Medical tourism companies conduct much of their business using the internet [53-55]. Reliance on the internet as a medium for communication, marketing, and interaction with clients means that much can be learned about medical tourism companies through careful analysis of their websites. Scholarly analysis of medical tourism companies should not be confined to content analysis of medical tourism company websites. Nonetheless, this mode of research makes an important contribution to the study of transnational medical travel.

#### Studying Inoperative Medical Tourism Companies

Finally, to date scholarship on medical tourism companies has examined companies currently engaged in the business of sending clients to international health care destinations. This article differs from previous work by identifying medical tourism companies that have ceased functioning. Identifying medical tourism companies that are inoperative raises questions about the nature and future direction of this industry. Many commentators assume that the medical tourism industry is in a period of unprecedented growth [56]. This article suggests that while the overall medical tourism industry might be expanding, the long-term success of individual medical tourism companies is not assured. The closure of twenty-five medical tourism companies in Canada might reflect a period of industry consolidation in which some companies flourish while others disappear or are subsumed by other companies. It is also possible that interest within Canada for travelling abroad to international medical facilities is not as large as is commonly assumed and these companies ceased functioning because many businesses are competing for a limited client base. Another possibility is that many of the companies erred when they based their operational model on sending clients to just one medical facility or country. It is conceivable that prospective clients want to have increased choice in where they travel for care and a business model that constrains options serves to deter clients that might otherwise consider going abroad for health care. And finally, since individuals considering going abroad for medical care can use the Internet to gain direct access to international hospitals and clinics, it is possible that medical tourism companies play a relatively minor role in the overall marketplace for medical travel. There might be less of a need for the service of medical travel facilitation than many entrepreneurs assume. Future research will explore in greater detail why the medical tourism companies identified in this study ceased functioning.

## Conclusions

This study identifies twenty-five defunct Canadian medical tourism companies. Researchers will likely disagree concerning how to interpret some aspects of these findings. Individuals supportive of the Canada Health Act and publicly funded access to medically necessary health services and critical of the privatized, for-profit health care marketed by medical tourism companies might argue that the failure of twenty-five medical tourism companies is a sign of limited interest within Canada for medical travel. They might claim that news media coverage of the phenomenon of medical tourism far outpaces the actual significance of the topic. In contrast, proponents of for-profit, private health care might assert that in every competitive marketplace some companies prosper while other businesses disappear. Whatever lessons can be extracted from the closure of twenty-five medical tourism companies established in Canada, it seems reasonable to suggest that establishing a medical tourism company in Canada provides no assurance of commercial success.

This study accomplishes several goals. Medical tourism companies use the internet to market health services to prospective clients. Their websites provide valuable information about where medical tourism companies are located within Canada, what countries and facilities they select as destination sites, what health services they advertise, how they position themselves in a competitive national and global marketplace, and what travel-related services they promote in addition to marketing health care. Content analysis of medical tourism company websites permits development of a detailed account of the current state of Canada's medical tourism industry. This article reviews defunct medical tourism facilitators in Canada. Websites of operational medical travel companies based in Canada are examined elsewhere. Various research methods and theories can be used to build upon the analysis provided here.

Many researchers lament the lack of empirical research into the study of medical tourism. This article addresses these concerns and opens new avenues for the study of medical tourism companies, medical travelers, and international health care destinations. It also reveals the importance of studying medical tourism in Canada and elsewhere, and not making the error of thinking that the medical tourism industry is primarily a product of the large population of uninsured individuals in the United States. Many researchers and other interested parties will likely be surprised to learn just how many medical tourism companies have emerged in Canada, a country where residents have publicly funded access to a large basket of medically necessary health services. They might be equally surprised to learn how many of these businesses have come and gone and no longer market transnational medical travel to Canadians. Establishing just how many residents of Canada use the services promoted by medical tourism companies based in Canada, examining what happens to medical travelers when they go abroad for care, and learning more about their experiences once they return to Canada will have to await additional studies by researchers from a variety of disciplines. This study attempts to make a substantial contribution to the study of the Canadian medical tourism industry. It also provides sign posts that should be of practical use to health researchers and other individuals interested in exploring ethical, legal, social, cultural, economic, and public health implications of Canada's medical tourism industry.

## Competing interests

The author declares that he has no competing interests.

## Author's contributions

LT is the sole author of this article. He designed the study, conducted content analysis of medical travel company websites, and wrote the manuscript.

## Author's information

The author studies ethical and social issues related to transnational medical travel and the emergence of a global marketplace in health services.

Leigh Turner

Associate Professor, Center for Bioethics and School of Public Health, University of Minnesota

N504 Boynton, 410 Church Street, Minneapolis, MN, U.S.A., 55455

Ph 612 626-4830

Fax 612 624-9108

## Supplementary Material

Additional file 1 **Medical Tourism Companies in Canada, Website URLs, and Webcited References**. This file lists in alphabetical order defunct medical tourism companies based in Canada, the website locations these companies used when operational (some links no longer function), and Webcite links that can be used to access archived websites. Click here for file

Additional file 2 **Core Marketing Messages of Canadian Medical Tourism Companies**. This file contains brief summaries of core marketing messages of defunct medical tourism companies based in Canada. It also contains excerpts of marketing messages found on websites of these companies. Click here for file
