# Supplementary material for: Canadian medical tourism companies that have exited the marketplace: Content analysis of websites used to market transnational medical travel
Source: Global Health. 2011 Oct 14;7:40. doi: 10.1186/1744-8603-7-40 (PMC3223128; doi:10.1186/1744-8603-7-40)
Supplement: Additional file 1 — Medical Tourism Companies in Canada, Website URLs, and Webcited References. This file lists in alphabetical order defunct medical tourism companies based in Canada, the website locations these companies used when operational (some links no longer function), and Webcite links that can be used to access archived websites. [file 1744-8603-7-40-S1.DOC]

**Additional file 1:**

**Medical Tourism Companies in Canada, Website URLs, and Webcited References**

| Axiom Health Solutions  <http://www.axiomhealth.org/>  Archived at: <http://www.webcitation.org/5xA4PbGje> |
| --- |
| Canadian Healthcare International (CHI)  <http://canadaheals.com/>  Archived at: <http://www.webcitation.org/5xA4b0hjf> |
| CubaMedicare  <http://www.cubamedicare.com/>  Archived at: http://www.webcitation.org/5zDve9Rel |
| EcuMedical Resources International Ltd.  <http://ecumedical.com/default>  Archived at: http://www.webcitation.org/5zDve9Rel |
| First Choice Medical Tourism  <http://www.firstchoicemedicaltourism.com/>  Archived at: <http://www.webcitation.org/5xA4pip0c> |
| Health Trips  <http://www.healthtrips.com/contact.htm>  Archived at: <http://www.webcitation.org/5xA4wjdfs> |
| Health Vacations, Inc.  <http://www.healthvacations.ca/>  Archived at: <http://www.webcitation.org/5xA54cVaN> |
| International Medical Network  <http://www.internationalmedicalnetwork.com/>  Archived at: http://www.webcitation.org/5zDve9Rel |
| JD Healthcare  <http://www.jdhealthcare.com/about.html>  Archived at: http://www.webcitation.org/5zDve9Rel |
| LAM-International (L.A.M. Logistic.Assistance.Medical International)  <http://www.laminternational.com/>  Archived at: http://www.webcitation.org/5zDve9Rel |
| MedAsia  <http://www.medasia.ca/>  Archived at: <http://www.webcitation.org/5xA5EonLp> |
| MedExpress Tourism  <http://www.medexpresstour.com/>  Archived at: http://www.webcitation.org/5zDve9Rel |
| Medi-Pro Medical Management  <http://www.medi-pro.org/>  Archived at: http://www.webcitation.org/5zDve9Rel |
| MedSolution  <http://www.medsolution.com/>  Archived at: <http://www.webcitation.org/5xA5Ks3P5> |
| Medtourlink  <http://www.medtourlink.com/>  Archived at: <http://www.webcitation.org/5xA5SCfzp> |
| Reach Health Services & Outsourcing  <http://www.reachhealthservices.com/>  Archived at: http://www.webcitation.org/5zDve9Rel |
| Recover Discover Healthcare  <http://www.recoverdiscover.com/>  Archived at: http://www.webcitation.org/5xA5ZhnN7 |
| Royal Med Services  <http://www.royalmedservices.com/>  Archived at: http://www.webcitation.org/5zDve9Rel |
| Speedy Surgery Global Healthcare  <http://www.speedysurgery.com/>  Archived at: http://www.webcitation.org/5zDve9Rel |
| Star Hospitals  <http://www.starhospitals.net/index.php>  Archived at: http://www.webcitation.org/5xA5qLRIP |
| Sun Medical Group <http://www.healthtourism.ca/>  Archived at: http://www.webcitation.org/5zDve9Rel |
| The InciDental Tourist  <http://www.incidentaltourist.com/>  Archived at: http://www.webcitation.org/5zDve9Rel |
| Tooth Tourism  <http://www.toothtourism.com/>  Archived at: <http://www.webcitation.org/5xA62hME4> |
| Unbelievable India  <http://www.unbelievableindia.com/>  Archived at: http://www.webcitation.org/5zDve9Rel |
| Victus Global Healthcare  <http://victus.ca/>  Archived at: http://www.webcitation.org/5xA67s2ve |
