# Supplementary material for: Canadian medical tourism companies that have exited the marketplace: Content analysis of websites used to market transnational medical travel
Source: Global Health. 2011 Oct 14;7:40. doi: 10.1186/1744-8603-7-40 (PMC3223128; doi:10.1186/1744-8603-7-40)
Supplement: Additional file 2 — Core Marketing Messages of Canadian Medical Tourism Companies. This file contains brief summaries of core marketing messages of defunct medical tourism companies based in Canada. It also contains excerpts of marketing messages found on websites of these companies. [file 1744-8603-7-40-S2.DOC]

**Additional file 2:**

**Core Marketing Messages of Canadian Medical Tourism Companies**

| **Company** | **Core Marketing Message: Summary and Excerpt** |
| --- | --- |
| Axiom Health Solutions | access to affordable, timely, and high-quality care  “Do you need urgent surgery but find your health plan can’t cover it? Are you compromising your life by delaying the medical treatment you really need? Are you on a waiting list to be painfree and stronger? **You can afford your surgery *now***…Let Axiom Health help you find top surgeons to perform your surgery at a fraction of the cost you would expect…Our partner facilities meet or exceed North American, British and European surgical techniques, equipment, devices and post-operative care.” |
| Canadian Healthcare International (CHI) | access to affordable and high quality health care in Canada  “Canadian Healthcare International (CHI) is an **international healthcare provider** committed to bringing the **standards and expertise of the Canadian Healthcare system to international patients**. The Healthcare in Canada is world renowned, but what is less commonly known is that the **cost of healthcare in Canada is significantly less than in the United States**, making Canada the trusted choice for your destination healthcare needs…Patients with various levels of insurance or no insurance at all will benefit from the cost savings of having their care in Canada, without compromising the level of care they receive.” |
| CubaMedicare | Access to affordable, timely, and high quality health care as well as vacation experience  “Our focus is in the field of Medical Tourism in Cuba. Our aim is to provide you with several options and information on medical facilities that are available on this Caribbean Island destination…Many medical tourists worldwide are seeking treatment at a quarter or sometimes even a 1/10 of the cost at home. From Canada, it is often people who are frustrated by long waiting times. For others, becoming a medical tourist is a chance to combine a tropical vacation with elective surgery…We have selected only a few medical procedures and Hospitals/Clinics in Cuba that offer international acclaim.” |
| EcuMedical Resources International Ltd. | access to timely and high-quality health care  “Are you in pain? Are you frustrated and angry with long wait times and minimal or restrictive medical options and treatments? Are you ready to take charge of your own health management? If so, we are here to help you get what you need and deserve! While we appreciate how great certain aspects of the Canadian health care system are, we are also acutely aware of its inherent limitations. While it is true that every Canadian gets free *basic* coverage, this type of system also means that lengthening wait times are inevitable and cutting-edge technologies are unaffordable…EcuMedical was passionately founded on a principle, on a vision to help all those who seek the best and fastest options available.” |
| First Choice Medical Tourism | access to affordable, timely, and high quality health care as well as holiday experience  “Overseas surgery is quickly becoming a safe and cost effective option to our healthcare here in Canada. There are many reasons why we should be traveling abroad for these services: high cost of health care services (dentistry for one); long waiting lists for surgery; ease and low cost international travel; good value for Canadian dollar in currency exchange rates; international accreditation of foreign hospitals; patient care that we often do do (sic) see any more; US, UK board certified surgeons operating in foreign countries…**First Choice Medical Tourism** specializes in **Dental, Optical, Cosmetic Surgery, Spa Retreats** and **Culture** in the **Philippines**. We provide you the opportunity to enjoy an **Exotic Holiday** and a **Medical Tourism Experience**.” |
| Health Trips | access to affordable and high-quality care as well as holiday experience  “Our goal is to connect people from Canada, USA, & England with the best **medical surgeries & treatments** at the lowest cost…Our office in India co-ordinates all your day-to-day needs and addresses all your concerns during your stay. We ensure that you will receive both a satisfying medical treatment and a relaxing holiday.” |
| Health Vacations, Inc. | access to affordable, timely, and high-quality care  “Our mission is to provide top-class medical tourism guidance with strong emphasis on our client’s safety and comfort…Whether you are insured or underinsured, Health Vacations Inc. can often find the right procedure for you at a fraction of the cost of what you would pay in Canada or the US. If you are on a waiting list or just want your procedure done quickly, we can usually have your procedure done in the hospital in a week. Medical tourism is growing at an explosive rate throughout the world. Health Vacations Inc. provides guidance for customers of medical tourism, finding high-quality, low-cost medical care in St. Petersburg, Russia.” |
| International Medical Network | access to affordable , timely and high-quality care  “International Medical Network is a private company established to assist in giving you options for medical care. IMN puts you in touch with international hospitals in order to save patients waittimes and money…International Medical Network insists that affordable, timely quality health care should be available to everyone.” |
| JD Healthcare | Access to affordable, timely and high-quality health care  “We have partnered with internationally renowned hospitals to give patients access to surgeries and healthcare for a fraction of the cost of what they would pay in their own country. ..Hospitals are frequently backlogged causing some operations to wait months or even years before a patient ever sees an operating table. In most cases, we can perform a surgery or operation in a few short weeks of your contacting us…JD Healthcare is your premiere source for affordable health care worldwide. Our patients come to us with their health requirements and we do the necessary preparation to get those needs taken care of in a safe, cost effective, and timely manner. The hospitals, doctors, and nurses we use have the same standards and practices as any of the top hospitals in the USA or UK.” |
| LAM International (Logistic.Assistance.Medical International) | Access to timely organ transplants  “LAM-International puts you in touch with professional health specialists formed in the best transplant centers in the world and always training in the latest techniques, updating their knowledge, skills and experience in the stages of pre organ transplant, during the organ transplant and after organ transplant; and providing a caring follow-up until referred back to your transplant doctors back at your home country. LAM-International makes it possible to put you on one of the shortest organ transplant waiting list times in the world!” |
| MedAsia | access to affordable, timely, and high-quality care  **“MedAsia is a health consulting office committed to enhance life quality of patients by offering an easy access to top-notch medical care in Asia. We give you access to Global Healthcare services outside Canada and the United States, so you can receive medical care at a fraction of the cost you would at home and no waiting list. MedAsia** gives you access to world-class healthcare through a comprehensive, patient-oriented process that will lead you to an improved life quality and achieve peace of mind.” |
| MedExpress Tourism | Access to affordable and high-quality care  “Getting world-class treatment at an affordable price should be everyone’s right. In that sense, Medexpress Tourism’s mission is to provide its patients access to a wide range of medical interventions at the best possible price and with the best quality care standards…MedExpress Tourism has its root in the simple evidence to help patients **get a good quality of care in Morocco**.” |
| Medi-Pro Medical Management | Access to timely and high-quality care  “**We help Canadians locate and secure the best healthcare options ‘On-Demand’. You demand immediate attention and we provide the options!** Instead of waiting in long queues in the public healthcare system or settling for a procedure that is outdated, we find cutting edge healthcare available immediately…**We have hospitals and clinics all over the US, many within driving distance. For specialized care we offer the best options around the world.”** |
| MedSolution | Access to affordable, timely and high-quality care  “MedSolution.com is a private company established to assist you in exploring your global health care options. We connect patients with international hospitals in order to save them money and time. We were created as a reaction to the exorbitant prices of health care in the United States and the increasingly long wait lists of Canada and the United Kingdom…MedSolution.com believes that quality, timely and affordable health care should be available to everyone.” |
| Medtourlink | access to high-quality care  “Medtourlink is designed to bring the most qualified and established businesses in the health and wellness sector together with consumers seeking health information and services…Our strategic partners are World Class JCI accredited hospitals in Singapore, Philippines, India, China, and Canada who are fully equipped with world-class medical facilities and technology where professionalism and safety is the top priority…For consumers, Medtourlink is to be the one stop source of information on providers and treatment packages, allowing people to search, find and connect with hospitals and doctors around the world. For doctors and hospitals, Medtourlink is an online platform to market their services to the millions of consumers searching the web for healthcare information. Using Medtourlink, patients are able to search and find doctors globally based on location, specialty, procedures and board certification, and treatment packages based on country and procedure.” |
| Reach Health Services & Outsourcing | Access to affordable and timely health care  “Medical tourism—Your immediate solution to Quick and Affordable Medical Procedures…RHS shall offer a complete package of surgical treatment in India for specialized healthcare sectors.” |
| Recover Discover Healthcare | access to affordable, timely, and high- quality care  “Recover Discover Healthcare has been helping people look for **Safe, High Quality & Economical Healthcare** options in India…We are honored and proud to stand by our tagline—**care-beyond-boundaries**...Recover Discover facilitates world class, high quality and affordable medical treatment in India. We also help clients experience the rich natural and cultural and historical facets of India.” |
| Royal Med Services | Access to high-quality health care  “Medical Treatment at World-Class Hospitals in India--& Elsewhere. Medical Treatment & tours to unbelievable exotic locations in India or elsewhere for an experience of a lifetime! Exacting Travel Arrangements & Personalized Health Care…*Every client is Royal, thus, we assure that you get the best out of each day on your trip*!” |
| Speedy Surgery Global Healthcare | Access to affordable, timely and high-quality health care  **“Access to highest quality healthcare in a speedy timeframe at an affordable cost**. Speedy Surgery is a global company, proud to play a central role in facilitating healthcare and providing healthcare information for our patients. Speedy Surgery facilitates access to services, ranging from diagnostic testing and everyday needs to the management of life threatening and critical illnesses. Speedy Surgery offers patient-centric services focused on providing information and access to healthcare through a global network of hospitals, physicians, clinics, and other healthcare institutions at volume discounted rates…Speedy Surgery has established a comprehensive network of health centers across Globe.” |
| Star Hospitals | access to affordable, timely and high-quality health care  “Star Hospitals offers patients a way to receive timely and affordable medical treatment. Our professionals will guide you through the whole process and stay in touch with you during your treatment and recovery…Star Hospitals is one of the leading health care facilitators in North America. Star Hospitals allows people to meet their medical needs while fulfilling their travel desires. We ensure only the highest quality of service.” |
| Sun Medical Group | access to affordable and timely care  “Welcome to Sun Medical Group Inc Health Tourism and Medical Tourism refers to the hundreds of thousands of patients that are currently traveling overseas for immediate and affordable treatment. This includes all types of surgery, in addition to cosmetic surgery, dental treatments, diagnostic or preventive health screening tests, or simply total Wellness Programs that will provide the best preventive health measure through a holistic approach. With substantially reduced treatment costs, most patients can afford to bring a companion and travel to an all-inclusive tropical resort for a recuperation period, close to your Doctors.” |
| The InciDental Tourist | Access to affordable and high-quality care  “The InciDental Tourist provides excellent dental and travel packages customized to meet your treatment requirements and, at the same time, treat you to an unforgettable guided visit that allows you to explore both the ancient and modern faces of China or the wonders of the Yucatan…The issue is **lower cost, not lower quality**…the money you will save on your dental procedures will more than pay for the cost of your vacation!” |
| Tooth Tourism | access to affordable and high-quality dental care  “Do you need a dentist but can’t afford the price? ToothTourism.com is here to make looking for dental treatments easy and *affordable*. When you compare local prices to clinics worldwide you see just how much money you can save—on the same quality dental work you receive at home. Dental tourism has helped many people realize their dreams of the perfect smile at a fraction of what they would spend locally!” |
| Unbelievable India | Access to affordable, timely, and high-quality care  “Medical tourism, the latest buzzword in travel & tour circles, is a reality in India…India offers highly specialized hospitals, medical professionals and expert postoperative care. You can gain easy access to state-of-the-art technology without putting a dent in your wallet. While hip replacement surgery would cost $15,000 or more in the US and other countries, the same procedure in India has about one-third the price tag. Also with increasing wait times under the Canadian, British and other government medical programs, it is no surprise that thousands of tourists are finding their remedy in India.” |
| Victus Global Healthcare | Access to affordable and timely health care (messages targeted at employers rather than individuals)  “Leading Canada in global healthcare…We provide your organization access to global healthcare. Two main advantages of global healthcare are the significant cost savings to your organization’s health benefits, and the on-demand medical care from a specialist for your employees…We have partnered with world-wide medical facilities that specifically offer international medical treatment. We then work with Canadian organizations and provide access to our partner network.” |
